# Morbidity through 3 Years of Age in Children of Women Using Methamphetamine during Pregnancy: A National Registry Study

**DOI:** 10.1159/000527238

**Published:** 2022-11-24

**Authors:** Roman Gabrhelík, Svetlana Skurtveit, Blanka Nechanská, Viktor Mravčík, Marte Handal

**Affiliations:** ^a^Department of Addictology, First Faculty of Medicine, Charles University, Prague, Czechia; ^b^Department of Addictology, General University Hospital in Prague, Prague, Czechia; ^c^Norwegian Institute of Public Health, Oslo, Norway; ^d^Norwegian Centre for Addiction Research at the University of Oslo, Oslo, Norway

**Keywords:** Prenatal exposure, Methamphetamine, Health registries, Child morbidity, Long-term effects, Hospitalization

## Abstract

**Background:**

There is a lack of studies on methamphetamine (MA) exposure and morbidity in children beyond the perinatal period.

**Objectives:**

We compared morbidity in children (0–3 years) with prenatal MA exposure to opioid-exposed and to non-exposed children.

**Methods:**

We used data from a Czech nationwide, registry-based cohort study (2000–2014). Children, who reached 3 years of age, of mothers hospitalized with (i) MA use disorder during pregnancy (MA; *n* = 194), (ii) opioid use disorder during pregnancy (opioids; *n* = 166), and (iii) general population (GP; *n* = 1,294,349) with no recorded history of substance use disorder (SUD). Information on inpatient contacts, length of stay, and diagnoses (International Statistical Classification of Diseases and Related Health Problems 10th Revision [ICD-10]) were assessed. Crude and adjusted odds ratios (aOR), 95% confidence interval (CI) for the risk of hospitalization, and for getting diagnosis from the ICD-10 diagnosis chapters were calculated using binary logistic regression. A stratified analysis on hospitalizations with SUD of mothers was performed.

**Results:**

No significant differences were found in the measures of hospitalization between the MA and opioid groups. Children prenatally exposed to MA and opioids had higher numbers of hospitalizations and diagnoses and longer stays in hospital than children in the GP. Increased risks of certain infectious and parasitic diseases were found in both MA (aOR = 1.6; CI: 1.1–2.3) and opioid (aOR = 1.9; 1.3–2.8) groups as compared to the GP group. The most pronounced difference in stratified analysis on maternal hospitalizations related to SUD after birth was observed for injury, poisoning, and certain other consequences of external causes in the strata of the MA group who had hospitalized mothers (aOR 6.3, 1.6–24.6) compared to the strata without maternal hospitalizations (aOR 1.4, 0.9–2.3).

**Conclusion:**

This study suggests that children born to mothers using MA during pregnancy have similar morbidity during the first 3 years of life but higher than the GP. The excess of risk was primarily due to infections and injuries in the MA group.

## Introduction

Methamphetamine hydrochloride (MA) use during pregnancy is increasingly common [1, 2, 3]. A considerable proportion of women do not seem to curb their MA use during pregnancy [4] despite the possible increased risk of adverse perinatal, neonatal, and childhood outcomes [5].

A limited number of studies analysed the association between prenatal MA exposure and the child's developmental outcomes beyond the perinatal period [6, 7, 8, 9, 10], and a majority focused primarily on behavioural, cognitive, and social problems in the children. The most rigorous study to date is the longitudinal Infant Development, Environment and Lifestyle(IDEAL) study in which 204 children with prenatal MA exposure and 208 unexposed were followed from delivery through childhood [11, 12]. This study did not find any associations between prenatal MA exposure and mental or psychomotor development in children at one, two, and 3 years of age [13]. In the MA-exposed children, only a subtle adverse effect on fine motor performance was observed at the age of one year, but this vanished in three-year-olds [13].

Although knowledge about long-term outcomes of prenatal exposure to illicit drugs is limited, an increasing number of studies report on the influence of factors such as drug-related lifestyle (e.g., relative poverty) on child outcomes [14, 15, 16]. However, no study has reported on the child's health condition requiring hospitalization beyond the perinatal period after prenatal MA exposure.

The aim of this study was to examine morbidity during the first 3 years of life among children with prenatal MA exposure. Specifically, we compared these children with children prenatally exposed to opioids as an attempt to control for unmeasured confounding since pregnant women using MA and those using opioids share similar background characteristics regarding socio-economic and lifestyle factors [16, 17]. This comparison also allows us to study whether the different substances used by the mother played an important role in the morbidity of children. Both groups were also compared to children of mothers from the general population (GP), i.e., without diagnosed substance use disorders (SUD) to study if there is any difference in the prevalence of morbidity between the two substance exposed groups and the unexposed. Based on our earlier results [17] showing worse neonatal outcomes in the opioid-exposed group than in the MA-exposed group, we hypothesized that the opioid-exposed children would have greater morbidity than children in the MA group. In addition, children from both the opioid and the MA groups would have a higher number of hospitalizations and increased morbidity than children from the GP.

## Methods

We linked data from Czech national health registries using the personal identification numbers [18] to investigate inpatient child morbidity.

### Data Sources

In Czechia, physicians are obligated by law to report data to the national health registries. The National Register of Reproduction Health (NRRH) holds information about maternal health; lifestyle during pregnancy; demographic and socio-economics; and information about delivery and the neonate, including birth parameters, congenital malformations, and death. The National Register of Addiction Treatment (NRAT) includes information about patients who receive opioids as addiction medication, e.g., date of initiation and termination of treatment and type of opioid.

The National Register of Inpatient Treatment (NRIT) provides information on all single hospitalization episodes, including dates of admission, discharge from hospital, and transfer to another department within the same hospital stay. The International Statistical Classification of Diseases and Related Health Problems 10th Revision (ICD-10) diagnostic codes were used in the discharge summary.

Hospitals represent the secondary healthcare level. The primary level is represented by the general practitioners for children and adolescents, and each child is registered to one specific general practitioner. The general practitioners must refer patients to the inpatient treatment. Outpatient emergency units in hospitals refer patients to inpatient departments. Nearly all hospitals have paediatric departments that provide acute care. The NRIT does not have information on patients who are only in contact with primary healthcare services. The Information System on Deaths (ISZEM) is a general mortality register providing records on time and cause of death for persons with a permanent or long-term residence in Czechia.

### Women Using MA during Pregnancy and Their Children

**Fig. 1:**
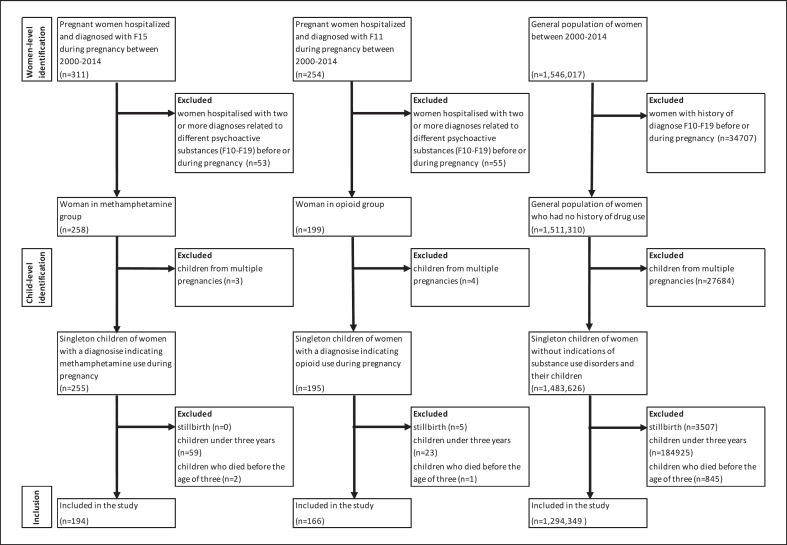
Flowchart on construction of cohorts of women and their children included in the study. Women-level identification. We identified women using MA and opioids during pregnancy and their children. The start and end of pregnancy data were retrieved from the National Register of Reproduction Health. Pregnant women who were hospitalized and diagnosed with mental and behavioural disorders due to use of other stimulants (ICD-10 code F15; all sub-codes registered in the National Register of Inpatient Treatment) during pregnancy were defined as women using MA during pregnancy. Women using opioids during pregnancy were defined as those hospitalized with a diagnosis of mental or behavioural disorder due to opioid use (ICD-10 code F11; all sub-codes). Children born to women with a diagnosis indicating opioid use during pregnancy formed the prenatal opioid group. Women using MA during pregnancy and women from the prenatal opioid group who were hospitalized with two or more diagnoses related to other psychoactive substances (ICD-10 codes F10-F19) before or during pregnancy were excluded. Women who were not diagnosed with any mental and behavioural disorders due to psychoactive substance use (ICD-10 codes F10-F19; all sub-codes) prior to or during pregnancy were defined as the GP of women who had no history of drug use (GP group). Women from the GP group who had a history any ICD-10 F10-F19 diagnose before or during pregnancy were excluded. Child-level identification: in all three groups, children from multiple births were excluded from the analysis. In all three groups, we also excluded (i) stillbirth; (ii) children who did not reach age of three years; (iii) children who died before the age of three years. Children who remained were included in the study.

The start and end of pregnancy data were retrieved from the NRRH. Pregnant women who were hospitalized and diagnosed with mental and behavioural disorders due to use of other stimulants (ICD-10 code F15; all sub-codes registered in the NRIT) during pregnancy were defined as women using MA during pregnancy since this diagnostic group is nearly exclusively represented by MA in Czechia (Fig. 1) [19, 20]. The diagnosis should reflect a relevant health problem of the patient for the actual hospital stay. Thus, to receive a F15 diagnosis during pregnancy, the woman should have used psychostimulants in pregnancy. Aside from less than 1% (*n* = 2) with an acute intoxication diagnosis, nearly all women in the MA group had a diagnosis indicating prolonged use. We excluded women hospitalized with two or more diagnoses related to different psychoactive substances (F10-F18) and women who were hospitalized for polydrug use (F19) before or during pregnancy in the study period to reduce the problem of polysubstance use (Fig. 1). Children born to women with a diagnosis indicating MA use during pregnancy formed the MA group. All children in this group were from single births.

### Women Using Opioids during Pregnancy and Their Children

Women using opioids during pregnancy were defined as those hospitalized with a diagnosis of mental or behavioural disorder due to opioid use (ICD-10 code F11; all sub-codes) [14]. Children born to women with a diagnosis indicating opioid use during pregnancy formed the prenatal opioid group. All children in this group were from single births (Fig. 1).

### Women without Indications of SUD and Their Children

Women who were not diagnosed with any mental and behavioural disorders due to psychoactive substance use (ICD-10 codes F10-F19; all sub-codes) prior to or during pregnancy were defined as the GP of women who had no history of drug use (GP group). Children from multiple births were excluded from the analysis (Fig. 1).

### Outcomes

Hospitalizations were chosen as the measure of morbidity. Information about hospitalizations of children was assessed for the time period from discharge from the hospital after birth until the age of three years. Data on all inpatient contacts from NRIT were used to assess information about length of stay and primary and secondary ICD-10 diagnoses (chapter level I–XXI) at discharge.

### Study Population and Study Period

The study population consisted of all children born in single births in Czechia during the study period, 2000–2014. Of these, 261 were children in the MA group and 197 were in the opioid group. Children of women from the GP with no recorded history of SUD formed the largest group (*N* = 1,495,370). Children who were born close to the end of the study period and therefore did not reach 3 years of age were excluded (Fig. 1). Also excluded were children who died before the age of three years (*N* = 2 [0.8%] in the MA group, *N* = 1 [0.5%] in the opioid group, and *N* = 845 [0.1%] in the GP group). The final study population consisted of 194 children in the MA group, 166 in the opioid group, and 1,294,349 in the GP group.

### Maternal Background Variables

Background characteristics of the pregnant women, such as age, marital status, education, previous abortions, smoking, and alcohol use during pregnancy, were obtained from the NRRH as described previously (18). These variables were chosen based on the literature suggesting the negative effect on birth outcomes and later child development [21, 22, 23]. We also used information on start of prenatal care and the number of medical controls during pregnancy as a proxy of lifestyle and health literacy characteristics [14, 16].

### Analysis Strategy and Statistics

Descriptive statistics (mean, median, and interquartile range) were used to present the proportion of hospitalized children, frequency of hospitalizations, length of hospital stay, and number of diagnoses (primary and secondary diagnosis) per child in each group who reached 3 years of age. Unadjusted and adjusted odds ratios (aOR) with 95% confidence interval (CI) for the risk of hospitalization were calculated by binary logistic regression and presented for the MA group compared to the opioid group and the MA and opioid group compared to the GP. Negative binomial regression analysis was used to calculate differences between groups in number of hospitalizations, length of stay, and number of diagnoses.

We then calculated the proportion of children hospitalized with different ICD-10 diagnoses during the period after discharge following birth until the age of 3 years. The population of children who reached 3 years of age was used as the denominator. CI for proportion was calculated using the continuity correlated score interval method (20). To control for relevant maternal background characteristics, we performed separate binary logistic regression for the categorical dependent variables (diagnoses yes/no) for each diagnosis chapter. Unadjusted and aOR with 95% CI were presented for the MA group compared to the opioid group and for the MA and opioid group compared to the GP. Significant results from the unadjusted analyses were adjusted for maternal age, marital status, education, smoking, alcohol use, and number of medical controls during pregnancy.

We also performed stratified analyses on (i) severe maternal substance use during the first 3 years after birth (yes/no) defined as a maternal hospitalization with an ICD-10 F10-F19 diagnosis within the child's first 3 years of life; (ii) premature birth (yes/no); and (iii) small for gestational age (yes/no).

The level of statistical significance for all analyses was set at *p* < 0.05 using 2-tailed comparisons. Statistical analyses were conducted using SPSS for Windows version 23 and Stata 14.

## Results

### Maternal Background Characteristics

Women from the MA group were younger and more frequently married compared to women from the opioid group (Table 1). As opposed to the GP group, both drug-related groups were younger, and most were not married. Among both MA and opioid users, more than half had only a primary education and a large proportion had previously had an induced abortion. The smoking prevalence was high in both drug-related groups. MA-using women started prenatal care two and a half weeks later and had fewer medical controls during pregnancy than those in the GP group.

### Hospitalization of Children

By the age of 3 years, 47.4% of children from the MA group and 53.0% of children from the opioid group had been hospitalized at least once, compared to 35.2% of children in the GP (Table 2). Children from both drug-related groups had higher numbers of hospitalizations and diagnoses and longer stays in hospital than children in the GP. Children in the MA group had slightly better, but not significant, outcomes as opposed to children from the opioid group regarding the number of hospitalizations, diagnoses, and length of stay.

After adjustment, the odds ratio (OR) of hospitalization was neither significant for the comparison between the MA and opioid group nor of the MA group versus the GP. Comparison between the opioid group and the GP was significant (aOR = 1.5; 95% CI: 1.1–2.0).

### Children's Diagnosis

Table 3 shows the proportions of children in the different groups based on the different diagnoses received until the age of three years. The most prevalent ICD-10 diagnostic chapters were chapters X Respiratory diseases (24.2% and 26.5%) and I Infections (18.6% and 22.3%) in the MA and the opioid groups, respectively. These two chapters were also among the most prevalent in the GP. The proportion of children with injury and poisoning (Chapter XIX) was 11.9% in the MA group; of these, 65% (*n* = 15) had head injuries (category S00-S09).

The unadjusted logistic regression analysis showed no statistically significant differences in the diagnostic chapters between the MA and the opioid groups (Table 3). In the unadjusted analysis when comparing the MA group to the GP, there were differences in risk for more than half of the diagnostic chapters. After the adjustment, the increased OR remained significant for certain infectious and parasitic diseases (aOR = 1.5; 1.0–2.2); diseases of the ear and mastoid process (aOR = 1.9; 1.1–3.4); certain conditions originating in the prenatal period (aOR = 1.8; 1.0–3.0); and injury, poisoning, and certain other consequences of external causes (aOR = 1.6; 1.1–2.5). Also, in the opioid group compared to the GP, increased risk of certain infectious and parasitic diseases (aOR = 1.9; 1.3–2.8) and certain conditions originating in the prenatal period (aOR = 1.8; 1.7–4.5) was observed (Table 3). There was no increased risk for injury, poisoning, or diseases of the ear when comparing the opioid group to the GP.

Stratified analysis on maternal hospitalizations related to SUDs during the first 3 years after birth showed tendency of higher ORs in all diagnostic categories compared to children of women without such hospitalizations (Table 4). The most pronounced difference was observed for the injury, poisoning, and certain other consequences of external causes in the strata of the MA group who had hospitalized mothers (aOR 6.3, 1.6–24.6) compared to the strata without maternal hospitalizations (aOR 1.4, 0.9–2.3). Results from the stratified analyses on premature birth and small for gestational age are presented in the online supplementary Table (for all online suppl. material, see www.karger.com/doi/10.1159/000527238). Stratified analyses showed mostly results in the same direction in both strata as in the main analysis.

## Discussion

We did not observe significant differences in morbidity in the children of women using MA during pregnancy compared to children of opioid using women during pregnancy, as measured by the hospitalization measures or the prevalence of ICD-10 diagnoses. By the age of three years, children in both the MA and the opioid group had higher risk of any hospitalization stay compared to the GP, though the risk did not remain significant for the MA group after adjustment. Other hospitalization measures such as the total number of hospitalizations and length of stay might still indicate more severe health conditions in the children in the MA and opioid groups compared to the GP. Children in the MA group received more diagnoses in several diagnostic chapters compared to the GP, but after adjustment, the increased risk remained significant only for the following diagnostic chapters: infectious and parasitic diseases; diseases of the ear and mastoid process; certain conditions originating in the prenatal period; and injury, poisoning, and certain other consequences of external causes. Adjustment for the socio-economic factors, illicit drug use, and the number of medical controls had a profound effect in all the comparisons with the GP.

To our knowledge, there is a lack of studies on MA exposure and morbidity in children beyond the perinatal period. There is also a paucity of data regarding other stimulants such as amphetamine, cocaine, and prescription of stimulant drug use [24].

The most pronounced differences observed were between the two drug-exposed groups and the GP. MA and opioids have different mechanisms of action, and both have undesirable outcomes for the child. One part of the explanation for these undesirable outcomes might be that the adverse effects can be linked to the drug using lifestyle common among both groups of pregnant women. This might be supported by the quite strong effect of adjustment for background characteristics and the number of controls during pregnancy seen in the analyses. The effect of socio-economic adjustment has been observed in studies of the association between several drugs and neonatal outcomes previously [16, 17]. In the stratified analyses in both the MA and opioid groups, we observed a tendency of higher risk estimates in the group where the mother was hospitalized for drug use drugs after birth. This similar result for the two drugs might also support the importance of lifestyle associated with drug use.

Irrespective of the reason for increased childhood morbidity, this study clearly showed that children in the two drug-exposed groups had markedly higher risk of hospitalization and on average two times longer hospitalization stays than children in the GP. For many diagnostic chapters studied, the proportion of children was higher in both drug-exposed groups than in the GP. This increase in morbidity is an important finding as it indicates that these children would benefit from close health care follow-up during childhood.

In a previous study, we showed that when MA- and opioid-exposed newborns were compared, some neonatal outcomes were more favourable in the MA-exposed [17]. In this study, no significant differences in childhood morbidity between MA- and opioid-exposed children were found. Nevertheless, all the hospitalization measures tended to be more favourable in the MA-exposed children when compared to the opioid-exposed. The same was observed for most diagnostic chapters.

When we compare the MA group with the GP, child injuries were diagnosed more frequently in the MA-exposed group. This was shown in both strata of maternal substance-related hospitalizations, but we observed higher risk in the strata where the mother was hospitalized after birth. This may refer to the negative role of the chaotic lifestyle of mothers and insufficient childcare that may subsequently lead to an increased risk of injuries, especially in women who use MA. This finding further supports our previous findings that increased peri- and postnatal morbidity of children could be linked to the lifestyle and socio-economic situation of their mothers [14, 17].

The higher risk of congenital malformations in children in the opioid group found in our study highlights the potential teratogenicity of opioids. According to a recent systematic review [25], positive associations were found between congenital malformations and maternal opioid use during pregnancy in more than half of the studies included. Nevertheless, most of the included studies had poor control over possible confounders including other teratogenic substance use during pregnancy (such as alcohol), which might also explain part of the teratogenic effect seen in our results.

### Methodological Considerations

We created a national cohort using longitudinal data from national registries of reproductive health, hospitalization, and death. Selection bias is therefore diminished relative to many other clinical samples. Such national registries generate larger samples than those that may practically be used in clinical studies. Furthermore, recall bias is also reduced with the inclusion of registry data.

For nearly a half century, MA has remained the most commonly used illicit substance in Czechia [26] with a high proportion of intravenous MA users [19], while the use of other types of stimulants, such as amphetamine or cocaine, is of very low prevalence [27]. This makes it possible to use the ICD-10 diagnosis F15 (stimulants use) to identify MA users.

One limitation is our definition of women using MA and opioids during pregnancy. We may not have identified a cohort of all pregnant women who have used substances while pregnant, rather only those whose use resulted in hospitalization. We defined exposure as a diagnosis of SUD during pregnancy; yet, we may have identified only the most problematic users. This definition could result in some misclassification. However, in the case of an exposure with low prevalence, specificity has a greater effect on the underestimation of risk than does sensitivity [28]. By using our definition of MA use, we have minimized the number of truly unexposed patients in the exposed group. An additional limitation results from the registers reporting MA as the primary problematic drug, while other illicit drugs may have been used in combination with MA. We also lack information about timing, duration, or dose. We had no information on burden of disease in the father or in the mother prior and during pregnancy. Therefore, we did not have the possibility to adjust for this information in our analysis. Further, the results from the regression analysis only show associations and do not point to causal effects, and the results must be interpreted with caution.

According to general practice in the Czech Republic, it seems that it is more common to hospitalize children in the Czech Republic than in other western countries. Hospitalizations due to childbirth were not included. In general, when a patient/child is moved from one ward to another for different health conditions (new diagnosis) during one hospital stay, this is recorded as two separate hospitalizations in the Czech registers. This may contribute to higher number of hospital stays. Finally, results of the stratified analysis were affected by a low number in different strata.

## Conclusion

In this nation-wide cohort of children prenatally exposed to MA, the total morbidity rate during the first 3 years of life was not significantly different from morbidity in children prenatally exposed to opioids. Compared to children in the GP, it seems like MA-exposed children had higher risk of infections and injury, which may be associated with lower level of care due to socio-economic conditions and the potentially chaotic lifestyle of mothers. These findings need to be replicated in other countries, preferably in larger study samples.

## Statement of Ethics

The study protocol has been approved by the Ethics Committee of the General University Hospital, Prague, No. 89/14 Grant VES 2015 AZV 1. LFUK. Written informed consent from participants was not required as this study used third-party data derived from the state government registries and databases.

## Conflict of Interest Statement

Roman Gabrhelík is the shareholder of Adiquit Ltd., which is currently developing apps for addictions recovery. Nevertheless, no funding was related to this study, and the activities had no role in the study design or the data collection, analysis and interpretation of the data, writing the manuscript, or the decision to submit the paper for publication. The remaining authors have no conflicts of interest to declare.

## Funding Sources

The study was supported by the Ministry of Health of the Czech Republic, Grant No. 16-28157A; Charles University Institutional support ID: COP-Addictology; and the Norwegian Research Council, Grant No. 240197/H10.

## Author Contributions

Concept and design: Roman Gabrhelík, Svetlana Skurtveit, and Marte Handal. Acquisition, analysis, or interpretation of data: Svetlana Skurtveit, Blanka Nechanská, Roman Gabrhelík, and Marte Handal. Blanka Nechanská had full access to all the data in the study and takes responsibility for the integrity of the data and the accuracy of the data analysis. Drafting of the manuscript: Roman Gabrhelík and Svetlana Skurtveit. Critical revision of the manuscript for important intellectual content: Roman Gabrhelík, Svetlana Skurtveit, Marte Handal, and Viktor Mravčík. Statistical analysis: Blanka Nechanská and Svetlana Skurtveit. Obtained funding: Roman Gabrhelík, Blanka Nechanská, Viktor Mravčík, Svetlana Skurtveit, and Marte Handal. Administrative, technical, or material support: Roman Gabrhelík. Supervision: Viktor Mravčík and Marte Handal.

## Data Availability Statement

This project uses third-party data derived from the state government registries and databases, which are ultimately governed by their Ethics Committees and data custodians. Thus, any requests to share these data will be subject to formal approval from each data source used in this project. Requests for data sharing/case pooling may be directed to the corresponding author's email: roman.gabrhelik@lf1.cuni.cz. Requests for code may be directed to Dr. Nechanská on email: nechanb@seznam.cz.

## Supplementary Material

Supplementary data

## Figures and Tables

**Table 1:** Socio-economic, drug use, and healthcare-related characteristics of pregnant women in Czechia, 2000–2014

	MA users (*n* = 194)			Opioid users (*n* = 166)			GP (*n* = 1,294,349)		
	*n*	%	95% CI	*n*	%	95% CI	*n*	%	95% CI
Age, years									
≤24	103	53.1	45.8–60.2	80	48.2	40.4–56.0	268,880	20.8	20.7–20.8
25–29	63	32.5	26.0–39.6	51	30.7	23.9–38.4	484,794	37.5	37.4–37.5
30–34	24	12.4	8.2–18.0	30	18.1	12.7–25.0	387,040	29.9	29.8–30.0
≥35	3	1.5	0.4–4.8	5	3.0	1.1–7.3	142,730	11.0	11.0–11.1
Marital status									
Not married	165	85.1	79.1–89.6	133	80.1	73.1–85.7	411,820	31.8	31.7–31.9
Married	25	12.9	8.7–18.6	27	16.3	11.2–23.0	853,567	65.9	65.9–66.0
Unknown	3	1.5	0.4–4.8	6	3.6	1.5–8.1	18,057	1.4	1.4–1.4
Education									
Primary	109	56.2	48.9–63.2	92	55.4	47.4–63.1	138,703	10.7	10.7–10.8
Secondary	76	39.2	32.3–46.5	67	40.4	32.9–48.3	877,972	67.8	67.8–67.9
Tertiary	0	0.0	0.0–1.9	2	1.2	0.2–4.7	207,414	16.0	16.0–16.1
Unknown	8	4.1	1.9–8.3	5	3.0	1.1–7.3	59,355	4.6	4.5–4.6
Abortions									
Induced	47	24.2	18.5–31.0	39	23.5	17.4–30.8	165,759	12.8	12.7–12.9
Spontaneous Using other substances during pregnancy	25	12.9	8.7–18.6	21	12.7	8.2–18.9	191,144	14.8	14.7–14.8
Alcohol (misuse)	6	3.1	1.3–6.9	10	6.0	3.1–11.1	1,533	0.1	0.1-0.−
Smoking	82	42.3	35.3–49.6	68	41.0	33.5–48.9	74,531	5.8	5.7–5.8
Deliveries by multiplicity									
Single	188	96.9	93.1–98.7	160	96.4	91.9–98.5	1,270,180	98.1	98.1–98.2
Twins and more	6	3.1	1.3–6.9	6	3.6	1.5–8.1	24,169	1.9	1.8–1.9

	mean	SD		mean	SD		mean	SD	
Start of prenatal care (weeks)	12.7	9.0		11.7	8.2		10.3	3.9	
Medical controls, *n*	7.0	5.4		6.8	4.9		11.4	3.6	

MA users − women hospitalized with a diagnosis of mental or behavioural disorder due to use of amphetamines (ICD-10 code F15, all sub-codes) during pregnancy. Opioid users − women hospitalized with a diagnosis of mental or behavioural disorder due to opioid use (ICD-10 code F11, all sub-codes) during pregnancy. GP − women who had no history of drug use defined as women who were not diagnosed with any of mental and behavioural disorders due to psychoactive substance use (ICD-10 codes F10-F19; all sub-codes) prior or during pregnancy. Education primary − consists of nine grades. Education secondary − 2− or 3-year course (vocational school) or 4-year course (professional school and lyceum). Education tertiary − higher professional school and university. CI, confidence interval.

**Table 2:** Hospital admissions in children (0–3 years) of women hospitalized with a diagnosis of mental or behavioural disorder in MA, opioid, and general population (GP) groups in Czechia, 2000–2014

	MA	Opioids	MA versus opioids (reference)	GP	MA versus GP (reference)^a^	Opioids versus GP (reference)^a^
Children who reached age of 3 years, *n*	194	166		1,294,349		
	
			OR (95% CI)		OR (95% CI)	OR (95% CI)

Hospitalized children, *n* (%, CI)	92 (47.4; 40.3–54.7)	88 (53.0; 45.1–60.7)	0.8 (0.5–1.2)	456,207 (35.2; 35.2–35.3)	**1.7 (1.3–2.2)**	**2.1 (1.5–2.8)**
	
			aOR (95% CI) 0.8 (0.5–1.2)		aOR (95% CI) 1.2 (0.9–1.6)	aOR (95% CI) **1.5 (1.1−2.0)**

			*P*		*P*	*P*

Number of hospitalizations, mean, median, IQR	2.1, 1.0, 1.0–3.0	2.4, 2.0, 1.0–3.0	0.288	1.8, 1.0, 1.0–2.0	**0.020**	<**0.001**

Length of stay in days, mean, median, IQR	15.1, 7.0, 4.0–17.0	18.1, 11.5, 4.0–23.5	0.241	8.4, 4.0, 2.0–8.0	<**0.001**	<**0.001**

Number of all diagnoses mean, median, IQR	4.1, 3.0, 2.0–5.0	4.7, 3.0, 2.0–6.0	0.185	3.0, 2.0, 1.0–4.0	<**0.001**	<**0.001**

Excluded: ICD-10 codes Z37 and Z38 diagnoses and birth hospitalization. MA- children of women hospitalized with a diagnosis of mental or behavioural disorder due to other stimulant use (ICD-10 code F15, all sub-codes) during pregnancy. Opioids − children of women hospitalized with a diagnosis of mental or behavioural disorder due to opioid use (ICD-10 code F11, all sub-codes) during pregnancy. GP-children of women who had no history of drug use defined as women who were not diagnosed with any of mental and behavioural disorders due to psychoactive substance use (ICD-10 codes F10-F19; all sub-codes) prior or during pregnancy. OR (95% CI) − odds ratios (ORs) from binary logistic regression of the child being hospitalized. We compared the MA and opioid groups, and the opioid group was the reference group. In the comparison between MA or opioid group with the GP, the GP was the reference group. aOR (95% CI) − adjusted odds ratios (ORs) for maternal age, education, smoking status during pregnancy, alcohol, and number of control, *p − p* value from negative binomial regression analyses. Cl, confidence interval. IQR, interquartile range.

**Table 3:** Binary logistic regression comparing children (0–3 years) of women hospitalized with a diagnosis of mental or behavioural disorder in methamphetamme (MA), opioid, and general population (GP) groups in Czechia

Chapter of ICD-10 diagnoses	MA (*n =* 194)	Opioids (*n* = 166)	GP (*n =* 1,294,349)	MA versus opioids (reference)	MA versus GP (reference)		Opioids versus GP (reference)	
	cases *n* (%)	cases *n* (%)	cases *n* (%)	OR unadjusted (95% CI)	OR unadjusted (95% CI)	aOR (95% CI)	OR unadjusted (95% CI)	aOR (95% CI)
I. Certain infectious and parasitic diseases (A00-B99)	36(18.6)	37 (22.3)	114,133 (8.8)	0.8 (0.5–1.3)	**2.4 (1.6−3.4)**	**1.5 (1.0−2.2)**	**3.0 (2.1−4.3)**	**1.9 (13−2.8)**
III. Diseases of the blood and blood-forming organs and certain disorders involving the immune mechanisms (D50-D89)	9 (4.6)	12 (7.2)	34,907 (2.7)	0.6 (0.3–1.5)	1.8 (0.9–3.4)		**2.8 (1.6−5.1)**	
IV. Endocrine, nutritional, and metabolic diseases (E00-E90)	13(6.7)	13 (7.8)	52,978 (4.1)	0.8 (0.4–1.9)	1.7 (1.0–3.0)		**2.0 (1.1−3.5)**	
VII. Diseases of the eye and adnexa (H00-H59)	7 (3.6)	7 (4.2)	18,408(1.4)	0.9 (0.3–2.5)	**2.6 (1.2−5.5)**		**3.1 (1.4−6.5)**	
VIII. Diseases of the ear and mastoid process (H60-H95)	14(7.2)	6 (3.6)	27,758 (2.1)	2.1 (0.8–5.5)	**3.5 (2.1−6.1)**	**1.9 (1.1−3.4)**	1.7 (0.8–3.9)	
X. Diseases of the respiratory system (J00-J99)	47 (24.2)	44 (26.5)	207,153 (16.0)	0.9 (0.6–1.4)	**1.7 (1.2−2.3)**		**1.9 (13−2.7)**	
XI. Diseases of the digestive system (K00-K93)	19(9.8)	22 (13.3)	78,546 (6.1)	0.7 (0.4–1.4)	**1.7 (1.0−2.7)**		**2.4 (13−3.7)**	1.5 (1.0–2.4)
XII. Diseases of the skin and subcutaneous tissue (L00-L99)	8(4.1)	5 (3.0)	36,147(2.8)	0.7 (0.3–1.7)	1.5 (0.7–3.0)		**2.2 (1.2−4.2)**	
XIV. Diseases of the genitourinary system (N00-N99)	7 (3.6)	8 (4.8)	46,102 (3.6)	0.7 (0.3–2.1)	1.0 (0.5–2.2)		1.4 (0.7–2.8)	
XVI. Certain conditions originating in the perinatal period (P00-P96)	15(7.7)	20(12.0)	35,721 (2.8)	0.6 (0.3–1.2)	**3.0 (1.7−5.0)**	**1.8 (1.0−3.0)**	**4.8 (3.0−7.7)**	**1.8 (1.7−43)**
XVII. Congenital malformations, deformations, and chromosomal abnormalities (Q00-Q99)	8(4.1)	14(8.4)	44,362 (3.4)	0.5 (0.2–1.1)	1.2 (0.6–2.5)		**2.6 (13−43)**	**1.9 (1.1−33)**
XVIII. Symptoms, signs, and abnormal clinical and laboratory findings, not elsewhere classified (R00-R99)	24 (12.4)	25 (15.1)	122,504 (9.5)	0.8 (0.4–1.5)	1.4 (0.9–2.1)		**1.7 (1.1−2.6)**	
XIX. Injury, poisoning, and certain other consequences of external causes (S00-T98)	23 (11.9)	12 (7.2)	82,637 (6.4)	1.7 (0.8–3.6)	**2.0 (1.3−3.0)**	**1.6 (1.1−2.5)**	1.1 (0.6–2.1)	
XXI. Factors influencing health status and contact with health services (Z00-Z99)	18(9.3)	22 (13.3)	58,730 (4.5)	0.7(03–1.3)	**2.2 (1.3−3.5)**		**3.2 (2.1−5.0)**	**2.3 (1.5−3.6)**

MA − children of women hospitalized with a diagnosis of mental or behavioural disorder due to MA use (ICD-10 code F15, all sub-codes) during pregnancy. Opioids − children of women hospitalized with a diagnosis of mental or behavioural disorder due to opioid use (ICD-10 code F11, all sub-codes) during pregnancy. GP − children of women who had no history of drug use defined as women who were not diagnosed with any of mental and behavioural disorders dueto psychoactive substance use (ICD-10 codes F10-F19; all sub-codes) prior orduring pregnancy. OR 95% CI -odds ratio with 95% confidence interval. Reference-in the binary logistic regression, when we comparing the MA and opioid groups, the opioid group was the reference group. In the comparison between MA or opioid group with the GP, the GP was the reference group. OR adjusted (95% CI) − aOR for maternal age, education, smoking status during pregnancy, alcohol, and number of control. Analyses were only performed for significant results from the first adjusted analysis.

**Table 4:** Binary logistic regression comparing children (0–3 years) of women hospitalized with a diagnosis of mental or behavioural disorder in methamphetamine (MA), opioid, and general population (GP) groups in Czechia, stratified on hospitalization (yes/no) of mother for F10-F19 during the first 3 years after childbirth

Chapter of ICD-10 diagnoses	Maternal hospitalization due to MA use after birth	
	yes	no
	*n* = 11 (MA), *n* = 1,492 (GP) OR adjusted (95% CI)	*n* = 182 (MA), *n* = 1,281,952 (GP) OR adjusted (95% CI)
I. Certain infectious and parasitic diseases (A00-B99)	3.5 (0.9–13.3)	1.4 (1.0–2.1)
VIII. Diseases of the ear and mastoid process (H60-H95)	4.6 (0.5–44.2)	**1.9 (1.1–3.4)**
XVI. Certain conditions originating in the perinatal period (P00-P96)	2.4 (0.3–20.5)	**1.7 (1.0–3.0)**
XIX. Injury, poisoning, and certain other consequences of external causes (S00-T98)	**6.3 (1.6–24.6)**	1.4 (0.9–2.3)

	Maternal hospitalization due to opioid use after birth	
	yes	no
	*n* = 4 (opioids), *n* = 1,492 (GP) OR adjusted (95% CI)	*n* = 162 (opioids), *n* = 1,281,952 (GP) OR adjusted (95% CI)
I. Certain infectious and parasitic diseases (A00-B99)	1.4 (0.1–13.8)	**1.9 (1.3–2.8)**
XVI. Certain conditions originating in the perinatal period (P00-P96)	5.7 (0.5–65.6)	**2.7 (1.7–4.4)**
XVII. Congenital malformations, deformations, and chromosomal abnormalities (Q00-Q99)	6.3 (0.6–69.5)	**1.8 (1.0–3.2)**
XXI. Factors influencing health status and contact with health services (Z00-Z99)	4.6 (0.4–48.3)	**2.2 (1.4–3.5)**

MA − children of women hospitalized with a diagnosis of mental or behavioural disorder due to MA use (ICD-10 code F15, all sub-codes) during pregnancy. Opioids − children of women hospitalized with a diagnosis of mental or behavioural disorder due to opioid use (ICD-10 code F11, all sub-codes) during pregnancy. GP − children of women who had no history of drug use defined as women who were not diagnosed with any of mental and behavioural disorders due to psychoactive substance use (ICD-10 codes F10-F19; all subcodes) prior or during pregnancy. OR 95% CI − odds ratio with 95% confidence interval. OR adjusted (95% CI) − in the binary logistic regression, when we compared the MA or opioid groups with the GP, the GP was the reference group. Adjusted for maternal age, education, and smoking status during pregnancy, alcohol, and number of control. Analyses were only performed for significant results from the first adjusted analysis.
