# Supplementary material for: Morbidity through 3 Years of Age in Children of Women Using Methamphetamine during Pregnancy: A National Registry Study
Source: Eur Addict Res. 2022 Nov 24;29(1):19–29. doi: 10.1159/000527238 (PMC9932820; doi:10.1159/000527238)
Supplement: Supplementary file 1 — Supplementary data [file ear-0029-0019-s01.docx]

Supplementary table. Binary logistic regression comparing children (0-3 years) of women hospitalized with a diagnosis of mental or behavioural disorder due to methamphetamine (MA), opioids and general population (GP) groups in Czechia stratified on child premature birth and small for gestatinal age.

|  | **MA versus GP (reference)** | | | | |
| --- | --- | --- | --- | --- | --- |
|  | **Premature** | |  | **Small for gestatinal age (SGA)** | |
| **Chapter of ICD-10 diagnoses** | **Yes** | **No** |  | **Yes** | **No** |
|  | **n=35 (MA), n=85062 (GP)** | **n=155 (MA), n=1198382 (GP)** |  | **n=23 (MA), n=49685 (GP)** | **n=167 (MA), n=1233759(GP)** |
|  | **OR adjusted (95% CI)** | **OR adjusted (95% CI)** |  | **OR adjusted (95% CI)** | **OR adjusted (95% CI)** |
| I. Certain infectious and parasitic diseases (A00-B99) | 1.2 (0.5-3.0) | **1.5 (1.0-2.3)** |  | 0.7 (0.2-2.5) | **1.6 (1.1-2.4)** |
| [VIII. Diseases of the ear and mastoid process (H60-H95)](http://apps.who.int/classifications/icd10/browse/2010/en#/VIII) | 0.6 (0.1-4.2) | **2.4 (1.4-4.3)** |  | 2.3 (0.7-7.8) | **1.9 (1.0-3.5)** |
| XVI. Certain conditions originating in the perinatal period (P00-P96) | 2.0 (0.9-4.3) | 1.5 (0.6-3.3) |  | 2.3 (0.9-6.4) | 1.5 (0.8-2.9) |
| XIX. Injury, poisoning and certain other consequences of external causes (S00-T98) | 2.2 (0.8-5.6) | 1.6 (1.0-2.6) |  | 1.3 (0.3-5.7) | **1.7 (1.1-2.8)** |
|  | **Opioids versus GP (reference)** | | | | |
|  | **Premature** | |  | **SGA** | |
|  | **Yes** | **No** |  | **Yes** | **No** |
|  | **n=36 (Opioids), n=85062 (GP)** | **n=127 (Opioids), n=1198382 (GP)** |  | **n=21 (Opioids), n=49685(GP)** | **n=142 (Opioids), n=1233759 (GP)** |
|  | **OR adjusted (95% CI)** | **OR adjusted (95% CI)** |  | **OR adjusted (95% CI)** | **OR adjusted (95% CI)** |
| I. Certain infectious and parasitic diseases (A00-B99) | 1.5 (0.7-3.4) | **2.0 (1.3-3.0)** |  | **2.7 (1.1-6.7)** | **1.7 (1.2-2.7)** |
| XVI. Certain conditions originating in the perinatal period (P00-P96) | 1.9 (0.9-4.0) | **2.8 (1.4-5.4)** |  | 2.2 (0.7-6.7) | **2.5 (1.4-4.4)** |
| XVII. Congenital malformations, deformations and chromosomal abnormalities (Q00-Q99) | 1.9 (0.7-4.0) | 1.9 (1.0-3.8) |  | 1.3 (0.3-5.6) | **2.1 (1.2-3.8)** |
| XXI. Factors influencing health status and contact with health services (Z00-Z99) | 1.2 (0.4-3.5) | **2.4 (1.4-4.0)** |  | 1.2 (0.3-5.3) | **2.2 (1.4-3.7)** |

MA - children of women hospitalized with a diagnosis of mental or behavioural disorder due to methamphetamine use (ICD-10 code F15, all sub-codes) during pregnancy.

Opioids - children of women hospitalized with a diagnosis of mental or behavioural disorder due to opioid use (ICD-10 code F11, all sub-codes) during pregnancy.

GP - children of women who had no history of drug use defined as women who were not diagnosed with any of mental and behavioural disorders due to psychoactive substance use (ICD-10 codes F10-F19; all subcodes) prior or during pregnancy.

OR 95% CI - odds ratio with 95% confidence interval.

(reference) - In the binary logistic regression, when we compared the MA or opioid groups with GP, GP was the reference group.

OR adjusted (95% CI) - Adjusted for maternal age, education and smoking status during pregnancy, alcohol, and number of control.
